# Influence of Probiotics on the Salivary Microflora Oral Streptococci and Their Integration into Oral Biofilm

**DOI:** 10.3390/antibiotics9110803

**Published:** 2020-11-13

**Authors:** Nicole B. Arweiler, Thorsten M. Auschill, Christian Heumann, Elmar Hellwig, Ali Al-Ahmad

**Affiliations:** 1 Department of Periodontology and Peri-Implant Diseases, Philipps-University, 35039 Marburg, Germany; auschill@med.uni-marburg.de; 2 Department of Statistics, Ludwig-Maximilians University, 80539 Munich, Germany; chris@stat.uni-muenchen.de; 3 Department of Operative Dentistry & Periodontology, Center for Dental Medicine, Faculty of Medicine, University of Freiburg, 79106 Freiburg, Germany; elmar.hellwig@uniklinik-freiburg.de (E.H.); ali.al-ahmad@uniklinik-freiburg.de (A.A.-A.)

**Keywords:** biofilms, probiotics, *Streptococcus mutans*, saliva, bacteria, oral microbiology

## Abstract

Probiotics’ ability to integrate into dental biofilms is not yet clarified. The aim of this trial was to detect probiotic bacteria from probiotic products in dental biofilm and saliva during and after intake. In this parallel, randomized clinical trial, 39 subjects wore customized appliances to build up intra-oral biofilms (72-h periods). The trial was divided into screening (S) to determine baseline biofilm flora, intervention (I), and wash out (WO). During I (28 days), subjects consumed a product containing (a) *Enterococcus faecalis* (b) *Lactobacillus* *casei*, or (c) *Lactobacillus rhamnosus* GG. Probiotic bacteria and Streptococci spp. were detected in the biofilms and saliva of the 35 subjects that were included in the analysis. During I and WO, the ratio of probiotics in the biofilm was very low compared to total bacterial load, while saliva had slightly but not significantly higher values. No significant changes of probiotic bacteria (*p* > 0.05) were found at any visit during I or WO. The proportion of streptococci was significantly reduced (*p* < 0.05) during I and even lower in WO, compared to S. Probiotic bacteria could neither integrate nor persist in dental biofilm and saliva but did influence the growth of streptococci in biofilm and saliva.

## 1. Introduction

The global probiotics market size was valued at 48.88 billion USD in 2019 and is projected to reach USD 94.48 billion USD by 2027, exhibiting a compound annual growth rate (CAGR) of 7.9% during the forecast period [1].

While probiotics have been praised for having a positive influence on the immune system and regarding some gut-related diseases [2,3], most health claims are still unsupported and systematic reviews reached no definite conclusions about their significant effects [4,5,6,7]. This is mostly due to the heterogeneity of yeasts, products, doses, and vehicles. The underlying mechanisms are seen in the competition with other yeasts, the interaction with virulence factors of other pathogens, the provision of nutrients and cofactors, and the stimulation of the host’s immune response.

Meanwhile, probiotics have also been scientifically evaluated according to their effect on oral health [8,9,10,11,12] because the oral cavity presents conditions similar to the gastrointestinal system regarding the high diversity of the microbiota and exposure to food components.

As to microbiome research in general, most research so far has focused on the gut microbiome [13], but recently the oral microbiome and its implication in oral [14] and systemic diseases [15] have been the subject of investigation. The oral microbiome is a highly dynamic community undergoing multiple changes in composition during childhood, with a more stable microbiota in adults. Dysbiosis characterized by overgrowth, e.g., by periopathogenic species, is seen as the cause of periodontal diseases like gingivitis and periodontitis, a chronic inflammation of the tooth-supporting structures with progressive alveolar bone loss [16]. Many studies have found clinical and inflammatory relationships between chronic periodontitis and other chronic metabolic, inflammatory, and vascular disease such as diabetes, cardiovascular diseases, chronic obstruction pulmonary disease (COPD), and metabolic syndrome and obesity [17,18,19,20]. Regarding caries, a further frequent oral disease, the total oral biofilm and especially oral streptococci play an important role as etiological agents [21].

Since dental diseases, especially periodontal diseases, are a major challenge to the immune system and general health [22], antipathogenic strategies are important pillars of prevention. Several agents, including antiseptics and antibiotics, have proven their preventive and therapeutic efficacy [23,24]. While studies have examined the effects of probiotic bacteria on salivary microbiota, gingivitis, and periodontitis [8,9,12,25], the underlying (explanatory) mechanisms of probiotic bacteria, such as their ability to integrate into dental biofilms resulting in displacement of other oral cavity bacteria, remain postulations and have only been shown in vitro [26,27,28]. So far, the few clinical studies on microbiological implication have only examined 10 subjects or less, were only of 14 days duration or applied probiotic bacteria only outside the mouth and only on glass slabs [29,30]. Thus, the aim of this randomized, three-armed clinical trial was to detect probiotic bacteria from probiotic products in in situ dental biofilms and saliva during and after 28 days of intake. The fate of the probiotics, as well as the proportion of oral streptococci, the major causative agents of caries, were examined in the supragingival biofilm and saliva using fluorescent in situ hybridization (FISH) and the culture technique.

## 2. Materials and Methods

### 2.1. Sample Size Calculation

Assuming α = 0.05, β = 0.2 and an effect size of 0.9, the paired Wilcoxon signed rank test (two-tailed) required a sample size of 13 (GPower 3.1.9.7).

### 2.2. Study Design and Population

**Figure 1:**
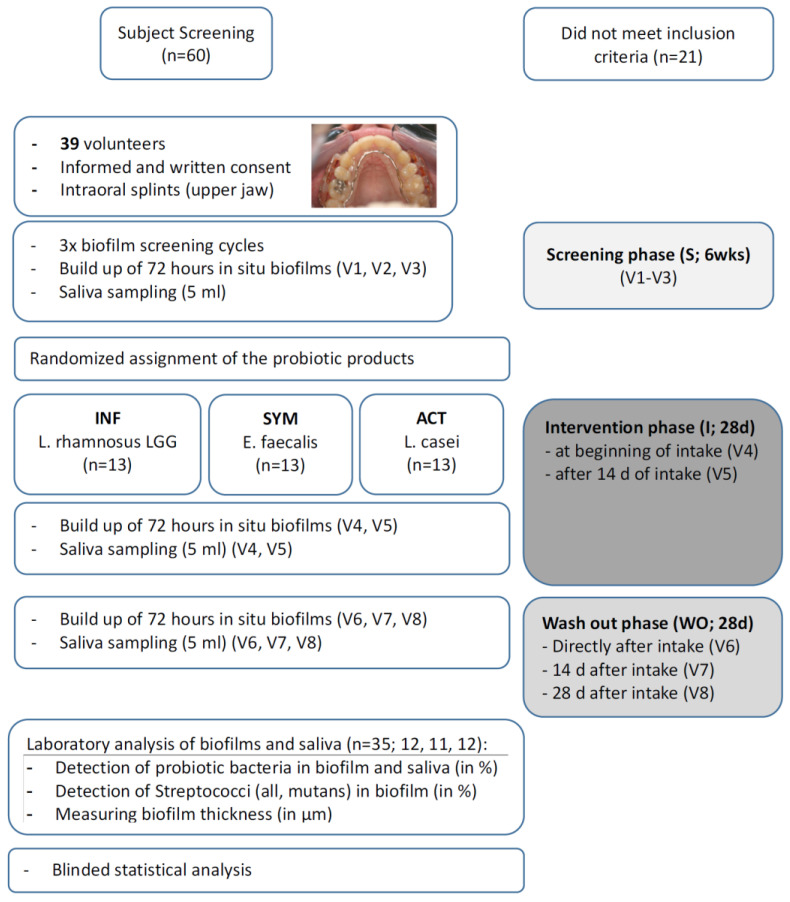
Flow chart of the study.

The observer-blind, parallel, randomized clinical trial took place at the Department of Periodontology and Peri-Implant Diseases of the Philipps-University, Marburg, Germany. It was approved by the Medical Ethics Committee of Philipps University (no. 44/11) and conducted in accordance with the ICH, Good Clinical Practice guidelines, and the Declaration of Helsinki (1964). The clinical trial was registered as ‘DRKS 00017822’. Sixty potential participants took part in an initial screening visit to assess their eligibility; the main inclusion criterion was a DMFT (number of decayed, missing, and/or filled teeth) of 0–5, corresponding to low caries experience. Patients had to be over 18 years of age, in good health, without any allergies to the products used in the study and had to be non-smokers (less than five cigarettes per day). They received participant information and signed an informed consent form. Exclusion criteria were any systemic diseases, smoking, untreated periodontal disease with signs of destructive periodontitis or inflammatory symptoms, caries, systemic antibiotic therapy within the last 6 months, a history of probiotic intake, or pregnancy. Furthermore, the use of any of the following drugs within the last 6 months: oral, intravenous, intramuscular, nasal or inhaled corticosteroids, and/or a history of viral infection (i.e., HBV, HCV, HIV) was excluded. In the end, 39 participants (aged 18–32 years) were included. A flow chart of the study is presented in Figure 1.

### 2.3. Grow-up of In Situ Biofilms

All volunteers received individually customized acrylic appliances for the upper jaw into which six plasma sterilized bovine enamel discs (diameter 3 mm, height 2 mm) were inserted. The three discs on each side were positioned towards the interdental area of two teeth in order to generate and mimic approximal biofilms (for details see [31] and Figure 1). A total of 1872 enamel discs were prepared for the eight wearing cycles. The appliances had to be worn continuously for 72 h (3 days) except during eating and oral hygiene measures (twice daily for 2 min each, using only an allocated toothpaste and toothbrush), during which the appliances were put into a dish filled with tap water.

### 2.4. Study Course and Products

The study was organized into a screening period (S), intervention period (I), and wash out period (WO). The screening period (S) consisted of three weekly visits (V1–V3), to determine the baseline biofilm and salivary flora. After S, the subjects were randomly allocated to the three different treatments (*n* = 13 each), according to a random allocation table and numbers assigned by the order of their attendance for the initial treatment phase. While the investigator and statistician were blinded, the participants were aware of the product since blinding of the products was not possible. The participants received the allocated product from a laboratory assistant not otherwise involved in the study. The randomization code was kept in a sealed envelope and was disclosed after all examinations and statistical analyses had been finished.

The intervention period (I) comprised two visits (V4: 72 h after beginning of intake, V5: after 14 days + 72 h of intake). During the 28 days of intervention, subjects had to consume one of the following products:

- Treatment 1: *Enterococcus faecalis* (SYM), Symbioflor 1 (SymbioPharm GmbH, Herborn, Germany); 30 drops, three times daily (=5 × 50 mL bottles);
- Treatment 2: *Lactobacillus casei* DN-114 001 (ACT), Actimel Classic (Danone GmbH, Munich, Germany); once daily, before lunch (=28 bottles during intervention);
- Treatment 3: *Lactobacillus rhamnosus* GG (INF); InfectoDiarrstop (InfectoPharm GmbH, Heppenheim, Germany), twice daily (morning and evening), diluted in water (=56 sachets).

All products were used or prepared according to the manufacturer’s instructions but had to be rinsed in the mouth for 2 min before swallowing, to allow a prolonged period of contact with the oral microbiota.

The wash out period (WO) consisted of three visits (V6–V8); the appliances were again worn directly after the last intake, after 14 and after 28 days, for 72 h each.

When the volunteers delivered their appliance at each visit, a 5 mL unstimulated saliva sample was collected in a centrifugation tube (Nunc IVF; ORIGIO GmbH, Berlin, Germany) and centrifuged with g = 1431 for 5 min (Labofuge 300, Kendro Laboratory Products GmbH, Langenselbold, Germany). Sampling of unstimulated saliva was chosen since oral microbiome analysis from unstimulated saliva performs equally to stimulated saliva or mouthwash rinse [32,33]. Supernatant was frozen immediately and stored at −80 °C in reduced transport fluid (RTF) [21]. The biofilm-covered enamel specimens were removed from the splints and processed for further analysis.

### 2.5. Fluorescence In Situ Hybridization (FISH)

The different probiotic bacteria, total oral streptococci, *Streptococcus mutans* and *Streptococcus sobrinus* were detected and their percentage compared to eubacteria, in biofilm and saliva, were calculated using the following specific gene probes, each on one enamel disc per subject.

- EUB 338 (5′-GCTGCCTCCGTAGGAGT-3′) for eubacteria (total count of all bacteria, [34]);
- LGC358a (5′-CCA TTG TGG AAG ATT CCC T-3′) for *Lactobacillus* spp. [35];
- Efs 129 (5′-CCCTCTGATGGGTAGGTT-3′) for *Enterococcus faecalis* [36];
- STR 405 (5′-TAG CCG TCC CTT TCT GGT-3′) for *Streptococcus* spp. [37];
- MUT590 (5′ ACT CCA GAC TTT CCT GAC-3′) for *S. mutans* [37,38];
- L-Ssob440-2 (5′ CAC ACG TTC TTC CCC TAC-3′) for *S. sobrinus* [35].

Briefly, the biofilms and saliva samples were fixed in 4% paraformaldehyde in phosphate-buffered saline (PBS, 1.7 mM KH_2_PO_4_—5mM Na_2_HPO_4_ with 0.15 M sodium chloride, pH 7.2) for 12 h at 4 °C. In order to minimize cell loss during the following hybridization and washing steps, the saliva samples were coated with agarose and spotted onto microscope slides (Erie Scientific Company, Portsmouth, NH, USA). Samples were further processed using FISH, as has been described in detail elsewhere [39].

### 2.6. CLSM Analysis

The labelled biofilms were visualized in a chambered cover glass (μ Slide 8 well; ibidi GmbH, Munich, Germany) with confocal laser scanning microscopy (Leica TCS SP2 AOBS, Mannheim, Germany) using a 63× water immersion objective (HCX PL APO lbd.BL 63.0 × 1.2W, Leica, Mannheim, Germany). The areas measured were from three representative locations on the biofilm and averaged for statistical analysis. The quantification of the different microbial targets within the biofilm (in %) and the measurement of biofilm thickness (BT in µm) were carried out as has previously been described [40].

### 2.7. Confirmation of Probiotic Bacteria by Culture Technique, MALDI-TOF and DNA Fingerprinting

In order to cultivate and identify the different probiotic bacteria *E. faecalis*, *L. casei* DN-114 001, and *L. rhamnosus* GG in the products, serial dilutions were incubated, and isolates analyzed and identified using MALDI-TOF-MS or the VITEK^®^2 microbial identification system and 16S rDNA gene sequencing. For details see [41]. To confirm the origin of the probiotic bacteria in the subjects’ biofilms and saliva from probiotic products supplied, pulsed-field gel electrophoresis (PFGE) was performed as described by Matushek et al. [42] and Klare et al. [43] with modifications as described by Al-Ahmad et al. [44]. The control strain *Staphylococcus aureus* NCTC 8325 was used as a molecular size standard for normalization.

### 2.8. Statistical Analysis

All parameters were analyzed for groups SYM, INF, ACT. The statistical analysis was conducted by C.H. using SAS. The intra group differences compared to screening were tested using the two-sided Wilcoxon signed rank test for paired samples (significance level α = 0.05). No multiple comparison adjustments were made.

## 3. Results

Of the 39 subjects, 35 (mean age 23.66 ± 3.44) completed all visits; in Group INF *n* = 12 (8 female, 4 male; age 23.17 ± 2.21), in group ACT *n* = 12 (4 female, 8 male; age 24.17 ± 4.61), group SYM *n* = 11 (6 female, 5 male: age 23.64 ± 3.29). One subject in the SYM group did not want to continue intake, three participants (one in each group) stated that they had underestimated the effort.

### 3.1. Detection and Percentage of Probiotic Bacteria by Multiplex FISH in Biofilm

**Figure 2:**
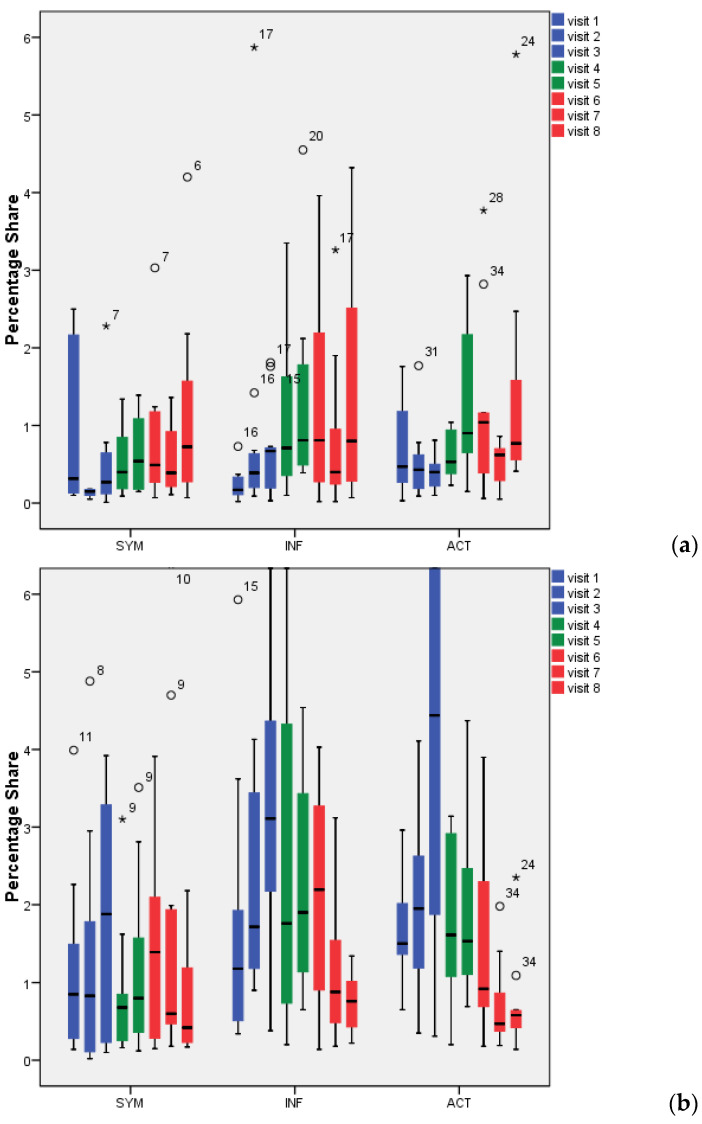
Percentage of lactobacilli/*Enterococcus faecalis* in 72-h dental biofilm (**a**) and saliva (**b**). °: Outliers with values between 1.5 and 3 times the interquartile range (IQR) above the 75%-quantile (Q3) (Q3 + 1.5 × IQR ≤ value ≤ Q3 + 3 × IQR); *: Extremes with values more than 3 times the IQR higher than Q3 (value > Q3 + 3 × IQR).

**Figure 3:**
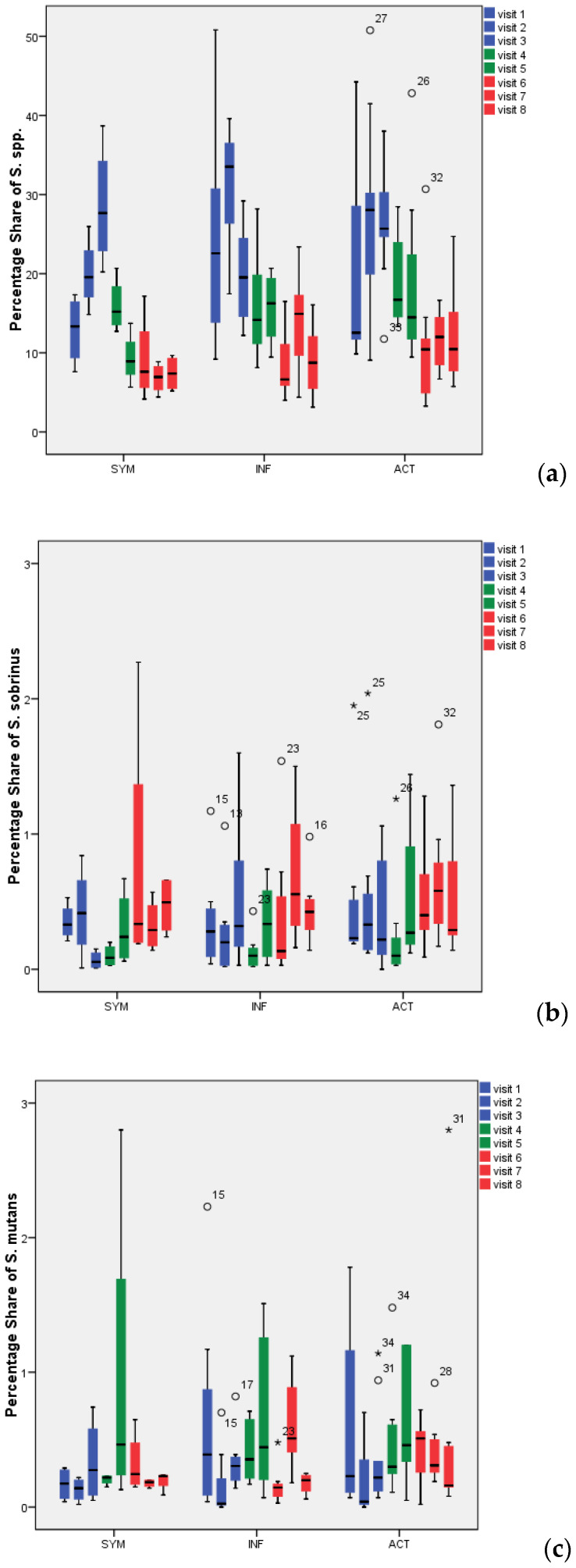
Percentage of all *Streptococcus* spp. (**a**), *Streptococcus sobrinus* (**b**), and *Streptococcus mutans* (**c**) in 72-h dental biofilm. °: Outliers with values between 1.5 and 3 times the interquartile range (IQR) above the 75%-quantile (Q3) (Q3 + 1.5 × IQR ≤ value ≤ Q3 + 3 × IQR); *: Extremes with values more than 3 times the IQR higher than Q3 (value > Q3 + 3 × IQR).

Figure 2a shows the percentage of probiotic bacteria (*Lactobacillus* spp. for treatments 1 and 2, treatment 3: *E. faecalis*) during screening (V1–V3; blue bars), during intake (V4, V5; green bars), and during wash out (V6–V8; red bars). The proportion of *Lactobacillus* spp. throughout the biofilm (see also CLSM images in Figure 3) was low for INF and ACT, with percentages not exceeding 2%. To compare the treatment products with the baseline, the screening visits (V1–V3) were averaged since they did not differ significantly (*p* > 0.05; Table 1). The I and WO percentages did not differ significantly within any of the treatment products compared to the baseline (*p* > 0.05). There were no significant differences between the three treatment groups (*p* > 0.05).

### 3.2. Detection and Percentage of Probiotic Bacteria by Multiplex FISH in Saliva

Saliva (Figure 2b) showed somewhat higher percentages of bacteria than biofilm (Figure 2a). *Lactobacillus* spp. were found at values of up to 4.5% (with the exception of V3 in ACT: 6.5%). While for the SYM group, no differences could be found between any of the visits (V4–V8) compared to baseline screening (*p* > 0.05), the ACT group revealed significant reductions in lactobacilli counts during visits 6, 7, and 8 (*p* = 0.034, 0.003, 0.003) and the INF group during visits 7 and 8 (*p* = 0.10, 0.002). Proof of the bacteria’s conformity during screening showed no match to the bacteria supplied. No significant differences could be found when the different groups were compared (*p* > 0.05).

### 3.3. Detection and Percentage of Streptococci by Multiplex FISH in Biofilm

**Figure 4:**
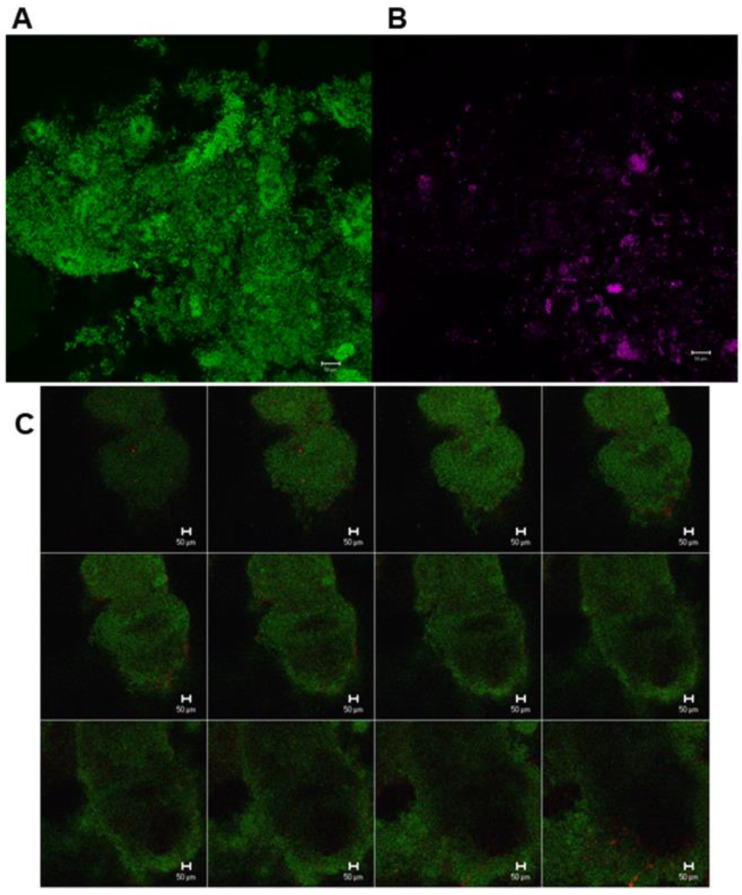
Confocal laser scanning microscopic (CLSM) images from different sections of in situ oral biofilm after the application of fluorescent in situ hybridization with different specific probes. (**A**) Green, eubacteria-specific; (**B**) *Streptococcus* spp.-specific probe; (**C**) Z-section galleries of representative CLSM images depicting a 3-day old biofilm hybridized with eubacteria-specific (green) and *Lactobacillus* spp.-specific probe (red).

The percentage share of *Streptococcus* spp., *S. mutans* and *S. sobrinus* in biofilm for all visits and all treatment groups is presented in Figure 3. Representative CLSM images for *Streptococcus* spp. are depicted in Figure 4. While SYM and INF groups showed significantly lower shares of *Streptococcus* spp. in all visits (V4–V8) compared to screening (*p*-values SYM V4:0.028, V5:0.008, V6:0.008, V7:0.011, V8:0.012; INF V4:0.003, V5:0.002, V6:0.002, V7:0.005, V8:0.003), reductions in ACT group were only significant for V6 (*p* = 0.01), V7 (*p* = 0.003), and V8 (*p* = 0.007) (Table 1). No significant reductions in *S. mutans* were found with the exception of the SYM group in V4 (*p* = 0.028), V5 (0.021), and V6 (0.038). *S. sobrinus* was not significantly reduced during these periods, with the only exception being V4 of the INF group (*p* = 0.008).

### 3.4. Culture Technique and DNA Fingerprinting

**Figure 5:**
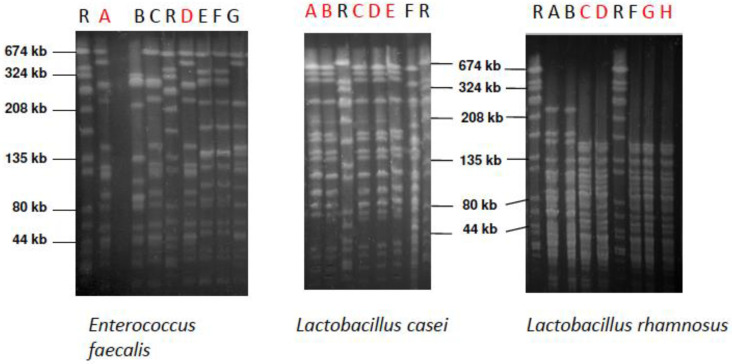
Pulsed-field gel electrophoresis (PFGE) patterns of *E. faecalis*, *L. casei*, and *L. rhamnosus* strains isolated from the supragingival biofilm of the different subjects. Red letters show the strains that were isolated from volunteers and genetically identical to the probiotic product strain. SYM strain: Lane A, ACT strain: Lane B, IINF strain: Lane H. Lane R above the PFGE indicates the reference strain *Staphylococcus aureus* NCTC 8325.

The culture technique used with MALDI-TOF and DNA fingerprinting with PFGE showed that most subjects’ biofilm and saliva did not include the original strain supplied during the different phases (S, I, WO) (Table 2, Figure 5).

An oral *E. faecalis* isolate was only detected in one subject, in low proportion in WO; this was revealed to be identical with the strain isolated from SYM. *L. rhamnosus* was detected in low concentrations in two subjects during S, in three subjects during I, and in two subjects during WO. However, only two *L. rhamnosus* isolates found during I and two during WO were confirmed to be similar to those supplied in INF. A few colonies of *L. casei* were found in one subject’s biofilm during S, for six subjects in I, and two subjects in WO. *L. casei* isolates from four subjects during I and two subjects during WO were revealed to be similar to those of ACT. All species were detected in most of the volunteers, at a low proportion of 10–10^3^ CFU/BES (bovine enamel slab).

*L. casei*, *L. rhamnosus,* and *E. faecalis* were also isolated in the saliva samples (Table 2). The concentration of *L. casei* in saliva was in the range of 30–10^5^ CFU/mL, whereas *L. rhamnosus* was detected at a concentration of 20–10^4^ CFU/mL. Only a few colonies of *E. faecalis* were revealed in saliva of one volunteer in S. *L. casei* was detected in two subjects’ saliva samples during S, four volunteers during I, and five subjects during WO. Only the *L. casei* isolates from three volunteers’ samples and only those collected during I, were similar to those isolated from ACT. *L. rhamnosus* was isolated from three volunteers’ saliva samples during S, seven subjects during I, and four during WO. The PFGE revealed similarity between the isolated *L. rhamnosus* from two subjects during I and the original strain isolated from INF, whereas such confirmation was only shown in two volunteers’ saliva samples during WO. *E. faecalis* was only detected in one subject during S but was not confirmed by PFGE.

Thus, when any lactobacilli or *E. faecalis* strains were found at all in FISH from I or WO, in biofilm or saliva, they were not derived from the products being tested, or were in negligible amounts, which supported the FISH conclusion (no significant increase due to intake).

### 3.5. Biofilm Thickness (in µm)

Mean thickness of the 3 day-old biofilms ranged between 11.00 (±3.04) and 20.85 (±7.71) µm in all treatments groups (Table 3) and did not change significantly between phases or treatment groups (*p* > 0.05).

## 4. Discussion

The hypothesis that probiotic bacteria are significantly raised during and after intake in biofilm and saliva was rejected, while the hypothesis that the number of *Streptococcus* spp. are significantly reduced during and after intake could be confirmed for SYM and INF. For ACT, *Streptococcus* spp. numbers were only significantly reduced after intake (WO).

A combination of two microbiological methods was applied to detect probiotic bacteria in biofilm and saliva. Due to the possibility of a diverse source of similar bacterial species, e.g., from food, the culture technique was still required in order to characterize and compare the strains isolated with the original strains cultivated from the probiotic products themselves by using PFGE [44]. The use of FISH in combination with confocal laser scanning microscopy allows for the integration of different probiotics within the biofilm to be explored in situ without its destruction [39,40]. Microbiome analysis using next-generation sequencing would have been beneficial for examining total variations in the oral microbiota; however, a specific examination of the probiotic bacteria—as in the present study—cannot be achieved.

In contrast to some in vitro or laboratory based studies, the present clinical study could not detect an increase of probiotic bacteria in dental biofilm even when supplied daily.

The clinical demonstration of the inability of probiotic bacteria to integrate into dental biofilm and saliva, either during or after 1 month of intake, confirms former statements (e.g., [12]) that probiotic bacteria cannot change adults’ residual oral microflora beyond the supply period. Teughels et al. [12] even recommend antibiotic therapy before probiotic supply, to improve their establishment in the microflora. This was not the case in the present study, which mimicked the normal situation when using commercially available probiotic preparations.

Lodi et al. [29] examined in a combined in vitro/in situ study the influence of two probiotic, fermented milk products, Batavito (B) and Yakult (Y), on biofilms, saliva microflora, and enamel. Ten subjects wore intraoral splints with enamel slabs for 14 days (on the palatal site) and either a 20% sugar solution or one of the probiotic products was applied eight times daily for 5 min outside the mouth and then the splints were worn again. After 7 days of wash out time, the other product was used (cross-over design). The biofilms were analyzed using classical cultivation on agar plates. Saliva was collected in a further experiment in which volunteers had to drink 80 g of one of the milk products. The authors could not detect any significant reduction in the total bacterial load (TBL) when comparing the probiotic to sugar application. The *S. mutans*, lactobacilli, and EPS (extracellular polysaccharides) concentration was similar in all the groups. Only in saliva was a significant reduction of TBL compared to the baseline seen, for B but not for Y.

An additional study using (similar to the present) a combination of CLSM and FISH analyzed a time series of samples from 6 to 72 h in two individuals and six participants that wore similar appliances (with glass slabs) for 48 and 72 h [30]. The individuals had to apply 5 mL of a probiotic milk product eight times a day (Cultura Dofilus^®^ naturell) on each side of the top of the integrated glass slabs. This short-term study used varying time points in their individuals, an extraoral application of the probiotics, no enamel slabs, and thus no strict study protocol as in the present study; therefore, no statistically supported statement was possible. However, they also could not detect any of three probiotic bacteria contained in the product in their in situ biofilms. In their saliva samples (although not strictly in all volunteers) probiotic bacteria were sporadically recorded.

The present study was also unable to detect significant differences between the I and WO periods, suggesting that these probiotic bacteria have no adherence or coaggregation properties in the oral cavity. Taking a mean salivary flow rate of 1 mL min^−1^ of the volunteers into consideration, a retention of probiotic bacteria would only be possible if they could adhere to hard tissues or could coaggregate with other supragingival biofilm microorganisms. So far, a coaggregation of *Lactobacillus salivarius* with oral streptococci could only be proven in few in vitro studies [28,45]. However, adherence properties of lactobacilli are currently highly discussed. Stamatova et al. [46] revealed high variations in the ability of different yoghurt starter lactobacilli to adhere to saliva-coated surfaces. In this context, the tongue, with its distinct surface characteristics (fissures, crypts, papillae, saliva), could also be prone to the colonization, growth, and proliferation of microbiota [47]. It is a highly populated niche which has a significant impact on the colonization of other regions in the oral cavity [48] and could be a noninvasive biomarker for oral and systemic health [49]. Since it could be expected that colonization of the tongue by probiotics may have an impact on the microbiota and the health of the oral cavity, the fate of probiotics within tongue coatings should be considered in future clinical studies.

In dentistry, such properties of probiotic bacteria could be both a blessing and a curse: many studies have shown changes in different cariological aspects after the supply of probiotic products, for example, in rats [50], in children [51,52], and in vitro (partly with human isolates; [10,11,27,29]). In their review, Gruner et al. [8] concluded that there was insufficient evidence for recommending probiotics for managing dental caries and refer to only short-term studies or parameters with limited value for the caries process (such as TBL, instead of caries incidence). Schwendicke et al. [10] highlight the principal cariogenicity of lactobacilli, as in their biofilm model they found significant mineral loss in dentin cavities caused by *L. rhamnosus* GG during sucrose supply concurrent with a lack of *S. mutans* inhibition. Such potential caries risk was taken into account by the study inclusion criteria of DMFT < 5 (decayed, missed, or filled teeth) in the study volunteers.

In contrast to the feared caries risks, a concomitant significant reduction in *Streptococcus* spp. share in 72-h biofilm was found during intake, which was even more pronounced during wash out. Thus, the data are in accordance with earlier studies that probiotic bacteria can replace or, better, suppress *Streptococcus* spp. [27,53,54]. Laleman et al. [9] could only include two studies in their meta-analysis, but confirmed that groups with probiotic intake showed significantly more subjects with lower streptococci counts and fewer subjects with higher streptococci counts compared to control groups. A very interesting and unique result from the present study is that the species *S. mutans* and *S. sobrinus* were in general not reduced by probiotic bacteria, while *Streptococcus* group and therewith obviously other species were significantly reduced by the probiotic supply. This seems to be in contrast to Laleman et al. [9], who found reductions in *S. mutans*. However, Laleman confirmed (in personal communication) that the commercial *S. mutans* tests which were used in the reviewed studies examined nonspecific counts of the *S. mutans* group and were not as specific as the present FISH technique which aims to detect *S. mutans* itself.

A recent study using high throughput sequencing analysis and the same splint system as the present study showed that the role and abundance of *S. mutans* in the in vivo supragingival oral biofilm is overestimated [21].

Such reductions can be explained by bacteriocins which are generally held responsible for effects in the gut and which could also have an influence on dental biofilm, without the direct integration of probiotic bacteria. Furthermore, Laleman et al. [9] did not find any differences between the intake and control group in terms of higher or lower lactobacilli counts, which is also in accordance with the present data.

So far, changes in biofilm thickness under the influence of probiotics have not been examined and are therefore not comparable. Significant reductions in biofilm thickness were only found in the SYM group (*E. faecalis*) at visits 4–6 (I and first WO visit). The lactobacilli supplied in INF and ACT were obviously not capable of reducing the biofilm thickness. It is interesting to note that, especially in these visits, SYM was also able to reduce *S. mutans* significantly.

## 5. Conclusions

Probiotic bacteria from all products were not able to integrate or persist in dental biofilm and saliva, but they did influence the growth of streptococci in biofilm. Biofilm thickness was not influenced (with the exception of *E. faecalis* during intake and early wash out). The interaction obviously does not happen through replacement but by interaction with other virulence factors, competition with nutrients or cofactors, or possibly, by stimulation of the host’s immune response. The present findings are initial steps to an explanation of the clinical effects of probiotic bacteria and products in oral cavity and further dental research.

The weak points of this clinical in situ study are intrinsically related to the small sample size and a targeted search for probiotic bacteria and oral streptococci, which could only provide an association and not determine a cause–effect relationship. Longitudinal studies with higher sample sizes and next-generation sequencing are required to examine the microbial fingerprint after (long-term) use of probiotics. Since oral bacteria are swallowed and translocated to the gut, affecting the intestinal health and disease [55], it is furthermore important to examine the influence of probiotic bacteria on both the oral and gut microbiota, which so far have not been studied in parallel.

## Figures and Tables

**Table 1:** Statistical analysis (*p*-value) of all parameters, comparing visits with screening period in the different groups.

Parameter (in Comparison to Screening)	SYM	INF	ACT
Biofilm			
% PB V4	0.477; n.s.	0.754; n.s.	0.347; n.s.
% PB V5	0.241; n.s.	0.286; n.s.	0.155; n.s.
% PB V6	0.477; n.s.	0.209; n.s.	0.286; n.s.
% PB V7	0.594; n.s.	0.530; n.s.	0.110; n.s.
% PB V8	0.959; n.s.	0.308; n.s.	0.239; n.s.
Saliva			
% PB V4	0.328; n.s.	0.433; n.s.	0.182; n.s.
% PB V5	0.534; n.s.	0.388; n.s.	0.091; n.s.
% PB V6	0.790; n.s.	0.272; n.s.	0.034; *
% PB V7	0.790; n.s.	0.010; **	0.003; **
% PB V8	0.142; n.s.	0.002; **	0.003; **
Biofilm			
% *Streptococcus* spp. V4	0.028; *	0.003; **	0.091; n.s.
% *Streptococcus* spp. V5	0.008; **	0.002; **	0.328; n.s.
% *Streptococcus* spp. V6	0.008; **	0.002; **	0.010; **
% *Streptococcus* spp. V7	0.011; *	0.005; **	0.003; **
% *Streptococcus* spp. V8	0.012; *	0.003; **	0.007; **
			
% *S. mutans* V4	0.028; *	0.754; n.s.	0.657; n.s.
% *S. mutans* V5	0.021; **	0.099; n.s.	0.286; n.s.
% *S. mutans* V6	0.038; *	0.099; n.s.	0.424; n.s.
% *S. mutans* V7	0.260; n.s.	0.114; n.s.	1.000; n.s.
% *S. mutans* V8	0.327; n.s.	0.110; n.s.	0.838; n.s.
			
% *S. sobrinus* V4	0.333; n.s.	0.008; **.	0.182; n.s.
% *S. sobrinus* V5	0.260; n.s.	0.695; n.s.	0.824; n.s.
% *S. sobrinus* V6	0.123; n.s.	0.583; n.s.	0.959; n.s.
% *S. sobrinus* V7	0.260; n.s.	0.114; n.s.	0.213; n.s.
% *S. sobrinus* V8	0.036; *	0.477; n.s.	0.314; n.s.
			
BT (in µm) V4	0.047; *	0.092; n.s.	0.575; n.s.
BT (in µm) V5	0.015; *	0.505; n.s.	0.131; n.s.
BT (in µm) V6	0.021; *	0.814; n.s.	1.000; n.s.
BT (in µm) V7	0.086; n.s.	0.959; n.s.	0.062; n.s.
BT (in µm) V8	0.401; n.s.	0.110; n.s.	0.374; n.s.

n.s.: non significant; *: *p* ≤ 0.05; **: *p* ≤ 0.01.

**Table 2:** Detected putative probiotic bacteria in biofilm and saliva samples in the different phase.

	*Lactobacillus casei*		*Lactobacillus rhamnosus*		*Enterococcus faecalis*	
	Participant #	Range/BES *	Participant #	Range/BES *	Participant #	Range/BES *
Screening (S) /Biofilm	1	10–500	2	10–40		
Ingestion (I)/Biofilm	6	20–700	3	10–100		
Wash out (WO)/Biofilm	2	500	2	20–100	1	2 × 10^3^
Screening (S)/Saliva	2	200–10^4^	3	40–1000	1	10
Ingestion (I)/Saliva	4	200–10^5^	7	100–10^4^		
Wash out (WO)/Saliva	5	30–2.2 × 10^3^	4	20–2000		

(#: number; * BES: bovine enamel slab).

**Table 3:** Biofilm thickness (BT in µm; mean ± SD).

	SYM	INF	ACT
Screening (V1–V3)	15.59 ± 5.41	15.54 ± 5.50	14.10 ± 4.54
Intake (V4)	11.00 ± 3.04	12.45 ± 3.34	13.03 ± 4.04
Intake (V5)	11.85 ± 5.44	14.89 ± 5.71	12.09 ± 5.42
Wash-out (V6)	11.18 ± 2.40	15.22 ± 6.57	13.82 ± 5.56
Wash-out (V7)	12.93 ± 5.05	15.93 ± 5.16	19.27 ± 6.77
Wash-out (V8)	16.75 ± 4.40	20.85 ± 7.71	18.76 ± 11.85
